# Suburban ethnicities: Home as the site of interethnic conviviality and racism

**DOI:** 10.1111/1468-4446.12738

**Published:** 2020-01-30

**Authors:** Katharine Tyler

**Affiliations:** ^1^ Department of Sociology, Philosophy and Anthropology University of Exeter Exeter United Kingdom

**Keywords:** British Asian Muslim Pakistanis, conviviality, home, racism, suburban, white working class

## Abstract

This article explores the ways in which the white working‐class residents of a suburban English town reflect on their relationships with their British Asian Pakistani Muslim neighbors. Its focus is on how everyday constructions of home become sites for the intermingling of discourses of intercultural conviviality *and* racism. My contention is that the idea of home has not yet been given the detailed critical attention that it deserves in the sociological literature on everyday manifestations of multiculturalism, conviviality, and racism. My supposition is that a special focus on the idea of home as the site of conviviality offers a productive avenue to analyze how intercultural relationships are formed and how the norms of neighborliness are thought to break down, opening a space for commonplace racialized and racist stereotypes to take hold. The idea of home is central to the rhythm and landscape of the English suburbs. It conjures up the idea of a uniform and aspirational white space. Drawing on this imaginary of home, I shall trace how “white working class” “English,” “Scottish,” and “Anglo‐Italian” residents’ everyday constructions of home become embroiled with their relationships with their British Asian Pakistani Muslim neighbors.

## 1 INTRODUCTION

Paul Gilroy (2004) famously deployed the concept of conviviality to capture and explain how ethnic and racial diversity is lived, experienced, and negotiated in multicultural London and other postcolonial cities. Gilroy’s focus is on how people discover that the things that they think might divide them come to co‐exist with an understanding of the things that they share in common. The concept of conviviality has also been widely mobilized by sociologists and anthropologists working in the fields of critical migration, race, and ethnicity studies (see Neal, Bennett, Cochrane, & Mohan, 2018, ch. 2 for an excellent overview). The emphasis of these studies is on how people live together across linguistic, religious, ethnic, racial, and national differences in super‐diverse cities. This includes studies focused on London (Back & Sinha, 2016; Wessendorf, 2013, 2014), Barcelona and Casamance (Heil, 2014), Singapore and Sydney (Wise & Velayutham, 2014), New York and Johannesburg (Nowicka & Vertovec, 2014), and cities across southern Europe such as Lisbon and Granada (Padilla, Azevedo, & Olmos‐Alcaraz, 2015). Read collectively these authors document and analyze practices of “everyday multiculturalism” (Wise & Velayutham, 2009), with a specific focus on the negotiation of cultural differences in “public” and “semi‐public” (Wise & Velayutham, 2014) spaces in urban settings, such as schools, cafes, parks, markets, leisure centres, and social clubs.

Crucial to this area of study is discussion of how the sometimes warm (Wise & Velayutham, 2014) or pragmatic (Wessendorf, 2013) relations of intercultural conviviality come to co‐exist with racist stereotypes, sentiments, and practices (Back & Sinha, 2016). The emphasis here is on the way in which wider postcolonial structures and social systems of difference inform everyday articulations of multiculturalism. In this vein, Back and Sinha (2016, p. 517) poignantly contend that the study of everyday multiculturalism must give equal weight to the “paradoxical co‐existence of both racism and conviviality.” Putting this the other way round, Wise (2015, p. 992 cited in Neal et al., 2018, p. 25) suggests that how “connection(s) to the concrete other start to reconfigure your view of the abstract other” is central to this arena of inquiry.

It has been acknowledged that the conviviality study’s approach to the sometimes “unruly” (Gilroy, 2004) aspects of everyday multiculturalism reveal that social policy models of multiculturalism are often overly simplistic, and blind to complexity (Kesten, Cochrance, Mohan, & Neal, 2011). Moreover, Neal et al. (2018) contend that conviviality studies contribute empirical details of everyday life that add concrete substance to abstract theories of multiculturalism. Yet in spite of these notable contributions, this area of study has also attracted criticism (e.g., Ahmed, 2010; Alexander & Nayak, 2016; Valentine, 2008). In their succinct summary of such criticism, Neal, Bennett, Cochrane, and Mohan (2019, p. 70) observe that the conviviality approach has been criticized for failing to deliver evidence of “meaningful interactions” that challenge racism. They also highlight the ways in which the conviviality studies’ picture of urban diversity has been critiqued for marginalizing “structural inequalities” and “the harms of racism” in an unthinking “celebration of diversity” (2019, p. 70), and so not seeing the realities of racism that shape everyday life.

My aim in this article is to contribute to thinking through a way that mediates between those who take the view that the conviviality studies’ approach idealizes intercultural conviviality at the expense of an analysis of racism, and those who are exponents of this approach. In so doing, my aim is not to adjudicate between these positions but to draw from both sides productively. To do this, I propose shifting the emphasis away from the study of public and semi‐public spaces as sites of conviviality‐and‐racism towards a focus on the more intimate site of the home. While scholars in conviviality studies do examine some of the ways in which home spaces are integral to practices of conviviality, they more often than not foreground other tropes of belonging. My suggestion is that this leaves the examination of home spaces as sites of conviviality‐and‐racism underexplored.

For example, Heil (2014) has studied ethnographically how Casamancais living in Catalonia and Casamance negotiate cultural differences. He explores the tensions and conflicts that arise among neighbors in communal home spaces when they think their shared norms of neighborliness have been breached. While home spaces feature centrally in this ethnography, the analytical focus is on the idea of *neighborliness* and not how everyday constructions of home explain difference. Similarly, Neal et al. (2018) examine young people’s—between 16 and 18 years of age—negotiation of cultural differences in educational spaces, showing how they reflect carefully on the ethnic constitution of friendship groups when considering who to invite to social gatherings at each other’s homes. Neal et al. (2018) also highlight some of the ways in which parental influences from home can shape who young people mix with at their colleges. But yet, as with Heil (2014), the analytical attention of these ethnographers also does not take in everyday constructions of home. That is to say, home space forms a backdrop to the analysis rather than being put at the center of the study and its conclusions. I might also mention my own analysis of what I call the “suburban paradox of conviviality and racism” (Tyler, 2016) which, while it explores how interethnic relationships are formed in home spaces, gives no special analytical attention to the ways in which ideas of home are constructed.

The exception here is Vincent, Neal, and Iqbal (2018, pp. 167–172) in their study of how social class and ethnic difference shape children’s (aged 8 to 9 years of age) friendships formed in primary schools based in super‐diverse areas of London. In contrast to the studies cited above, these scholars pay detailed critical attention to how children’s and their parents’ friendships, formed across ethnic, religious, and class differences, extend into the intimacy of home spaces. In this way, these scholars discuss how home spaces become sites for children and parents to consolidate, manage, and negotiate diverse ethnic, racial, religious, and class relationships formed at school. Thinking of the conviviality studies’ approach to diversity, these scholars poignantly write: “Putting home space as central to debates about cultural difference and affective relationships remains under‐researched and home tends to be seen as outside of or apart from the public and semi‐public spaces of encounter” (2018, pp. 167–168).

So in this article I seek to begin to redress the side‐lining of everyday constructions of home in previous studies on conviviality‐and‐racism. To do this, I shall put at the heart of my analysis an exploration of how home spaces become the sites for relations and interactions across perceived ethnic, national, and racial identities. My suggestion is that sustained focus on the idea of home as the site of intercultural conviviality affords a productive avenue through which to analyse how social norms of neighborliness are formed and sometimes break down across ethnic, national, and racial differences. Importantly, this will open up an analytical space for the exploration of how widely held racialized and racist stereotypes are reproduced.

To illustrate these processes, I draw on a qualitative study of white working‐class people’s experiences of ethnic, national, and racial diversity that I conducted with other researchers in a suburban town situated in the south‐east of England, to which I give the pseudonym “Southtown.”^1^ In the empirical sections of this article, my focus is on the ways in which white working‐class residents, across the national identities of Scottish, English, and Anglo‐Italian, describe their relationships with their British Asian Muslim Pakistani neighbors. But before turning to the details of this study, some reflection on the concept of *home* and how it is applied in my analysis is needed.

## 2 HOME, CONVIVIALITY, AND RACISM IN A SUBURBAN TOWN

Over a number of years my work on everyday multiculturalism has focused on sites that form a stark contrast to the cosmopolitan cities that usually feature in the literature on interethnic cultures of conviviality (Tyler, 2012, 2015, 2016). In this regard, my work contributes to a growing body of literature that examines the particular articulations of multiculturalism in smaller towns and cities in the UK that are not generally known for their ethnic diversity (e.g., Erel, 2011; Gidley, Hanson, & Sundas, 2018; Kesten et al., 2011; Neal, Bennett, Cochrane, & Mohan, 2013; Neal et al., 2018; Rogaly & Qureshi 2013). Given the particular significance of the idea of home to the imagery of English suburban life, it is not surprising that home emerged as the dominant site through which people spoke about their relationships across ethnic, national, and racial differences in our study of an English suburban town. The idea of the English suburban home evokes the image of a uniform and heteronormative secure and aspirational white space associated with classed notions of respectability (Huq, 2013). Recent studies of multiculturalism in smaller British towns and cities highlight the daily negotiation of white aspirational classed norms identified with the home spaces of suburban life. For example, in Erel’s (2011, pp. 2058–2059) study based in Peterborough, a town in the East Midlands area of England, the disposal of domestic rubbish and the concern to maintain public and private boundaries between neighbors become sites of racial tension for long‐term white British residents in their interactions with their migrant neighbors. Moreover, sociological and anthropological analysis of home and suburban life complicates any singular notion of these aspects of social life (e.g., Mallet, 2004; Huq, 2013). For example, the study of suburban life emphasizes the diverse, fluid, and cosmopolitan, socio‐economic constitution of the suburbs (Dwyer, Gilbert, & Shah, 2013; Huq, 2013). In this vein, Watson and Saha (2013) draw on Stuart Hall’s notion of “multicultural drift” to emphasize the “ordinary” and “subtle” ways in which ethnic and racial diversity is a fact of life in London’s predominantly white suburbs. Similarly, the anthropological and sociological study of home illuminates how the idea of home is a multidimensional concept and phenomenon (Ahmed, Castaneda, Fortier, & Sheller, 2003; Brah, 1996; Mallet, 2004). Thus everyday ideas of home encompass the affective and the material, the spatial and the temporal, the local and the global, the positively valued (e.g., warmth and security), and the negatively valued (e.g., suppression, inequality, and harm) (Mallet, 2004).

Poignantly for my exploration of how home becomes the locale for expressions of conviviality and racism, Brah (1996) has influentially argued that home is constituted by relations of power that serve to include and exclude. She writes: “[home is]…intrinsically linked with the way in which processes of inclusion and exclusion operate and are subjectively experienced” (1996, p. 192). Brah (1996, p. 192) suggests that home is “essentially about our political and personal struggles over the social regulation of ‘belonging’.” In short, for Brah (1996), home is a contested space that is the site for the reproduction of racial, ethnic, gender, class, and sexual relations of inclusion and exclusion that constitute identity.

Advancing Brah’s approach, Ahmed et al. (2003, p. 10) mobilize the concept of “home work” to explore how home can be a closed and bounded place, but also the site of hope, conviviality, and openness. They maintain:

Home work is affective work, but the nature of that work must be specified: it may be a labour of love, of hatred; it may involve conservative nationalist desires or claims to homelands as historical reparation; it may be haunted by fear or loss or filled with hope for different, more peaceful and equitable futures.

Drawing on the ethos and ethic of these approaches to the everyday construction of home, I shall trace how home becomes a place of emotional *security and stability* for the reproduction of self and collective identity in the narratives of white working‐class Scottish, English, and Anglo‐Italian residents that live in Southtown. I am inspired here by Skey’s (2011) study of everyday articulations of the nation as a homely, secure and familiar place. He examines how, for white English people living in England, the nation is constructed as home to the extent that it evokes a secure, familiar, and stable place from which to visit other places in the world and return to. I shall show how for the residents of Southtown, home is also constructed in diverse ways as a secure and familiar place that offers a sense of “ontological security” (Giddens, 1990 cited in Skey, 2011, p. 234). In the empirical sections of this article that now follow, I shall trace the varied norms and values that constitute this sense of homely stability, security, and familiarity. I will bring to the fore how these everyday constructions of home are put to work both to include and to distance and exclude those that are thought to be racially, ethnically, and nationally other to the white self.

## 3 FIELDWORK IN SOUTHTOWN

The research team, including myself, conducted fieldwork in Southtown between 2008 and 2009. This time is of socio‐political significance because it signaled the beginning of the most recent global economic crisis. It was also not long after the accession of the A8 countries to the EU in 2004 and the subsequent movement of migrants to the UK from Eastern Europe. While the government’s austerity agenda had not yet been implemented in the UK, the 2008 economic crisis combined with the increased migration of people from Eastern Europe to the UK are thought by some media commentators and academics, to be key events in informing the public’s negative attitude towards immigration (for a broader discussion see Kriesi et al., 2008). The latter in turn has been identified by the mainstream media, politicians, and social scientists as crucial to the outcome of the 2016 referendum on Britain’s membership of the European Union (e.g., Clarke, Goodwin, & Whiteley, 2017). From this point of view, my reflections on articulations of home, conviviality, and racism in Southtown offer some insight into the often complex and contradictory ways in which people living in Britain engaged with issues of multiculturalism and immigration before the Brexit referendum. This helps to contextualize its outcome and aftermath (see also Degnen & Tyler, 2017 for a similar argument in relation to the anthropology of Britain). Moreover, these data take on new significance when viewed through the lens of contemporary academic, media, and political Brexit‐laden narratives on race, immigration, class, and generation, a theme I shall return to below.^2^

Southtown is within commuting distance of London and is set in one of the wealthiest counties of England. Southtown has a population of approximately 100,000 inhabitants and 75 per cent of its residents according to the official government categories used in the 2011 Census (e.g., the most recent one to the time of the fieldwork) are “White: British.” However, the neighborhood in which our fieldwork was based is ethnically diverse. Only 37% of the total population of the fieldwork neighborhood identified as “White: British,” and 29% identified as “Other.” The largest ethnic minority in the fieldwork neighborhood according to the 2011 Census was people who self‐identified as “Pakistani” who made up 34% of the neighborhood. Moreover, “Muslims” identified as the largest religious group in the fieldwork neighborhood at 38% of the total population, while 34% self‐identified as “Christian.”

People from the Mirpur area of Pakistan settled into the neighborhood in the 1960s in the aftermath of British decolonization of India. One of the reasons that Muslims migrated to the neighborhood is its mosque. At the same time southern Italian migrants also settled in the area to mostly take up work on fixed‐term contracts on nearby farms. At the time of fieldwork British Italian residents estimated there were about 1,500 Italian‐descent families living in the town (see Fortier, 2000, pp. 26–31 for an overview of Italian migration to Britain). Since the turn of this century, the area has experienced the settlement of increasing numbers of migrants from across the world, most notably from Poland and Nepal. To summarize, Southtown is a predominantly white British town in terms of ethnic and national composition. However, the neighborhood in which our fieldwork was conducted is ethnically, racially, and nationally diverse.

Interestingly, given my focus on ideas of home, the fieldwork neighborhood showed patterns of home ownership and rental that were distinct from the rest of the town. The percentage of households that owned their own homes in the fieldwork neighborhood was 47% in 2011 (ONS cited in Jensen, 2013, p. 445); this is considerably less than the town as a whole, where 70% of households in the town owned their own homes. 29% of households in the fieldwork neighborhood rented from the local council or housing associations compared to 12% for the town as a whole. And 16% of housing was rented from private landlords in the fieldwork neighborhood compared to 8% for the town as a whole (see Jenson, 2013, for a detailed discussion of housing statistics in the town). In short, then, more than twice as many people rented houses in the neighborhood in which the fieldwork was based, and the proportion of home ownership was far lower than in the rest of the town.

The conversations with research participants that I shall draw on in this article formed part of 63 in‐depth informal conversational‐style interviews with a range of residents in terms of age, gender, religious, class, and ethnic identifications. The aim of the original research project was to explore how British Asian, white English and British Italian residents of this ethnically diverse neighborhood identified with their place, with each other and new migrants to the area. The empirical data included biographical interviews conducted with residents in families and across generations, as well as conversational‐style interviews about the socio‐economic constitution of the town and the neighborhood with local community workers. The interviews were digitally recorded and conducted by the research team that included a research fellow who permanently lived in the area, two community‐based researchers, one of whom already lived in the area and the other who worked for a local organization, and myself, the project’s principal investigator, who lived nearby.

In what follows, I have chosen to follow an established representational strategy in qualitative sociology and anthropology by drawing in detail on specific case studies (see, e.g., Back & Sinha, 2016 for a similar approach to writing about conviviality‐and‐racism in London). I shall concentrate in some depth on three cases drawn from different households involving four individuals. This representational strategy draws me close to the details of individuals’ accounts and allows me to enter their life‐worlds in a way that a thematic representation of the entire data‐set would not.

Given the qualitative nature of the study, I do not want to claim, as the author of a quantitative study might, that my analysis is representative of the wider research sample from which it is drawn. Nor do I want to give the impression that Southtown itself represents an archetypical suburban English town. Rather, what is reported in this article is a partial representation of a dominant thread that was woven throughout the entire interview data‐set: namely, that home was positioned as a reoccurring site for the articulation of discourses of interethnic conviviality‐and‐racism. I have purposefully chosen to focus on individuals that display from each other differing degrees of working‐class cultural and material capital in terms of occupational status, social networks, and home ownership. I draw mainly on the thoughts of older white residents who are in their fifties, sixties, and seventies. These individuals are long‐term residents to the town and have over time formed a deep sense of attachment to this place that becomes configured in their diverse constructions of home. I have also chosen the accounts of participants whose identities illustrate the national heterogeneity of white working‐class people’s identities that live in England. The individuals that feature in this article self‐identify as “Anglo‐Italian,” “Scottish,” and “English.” It is also worth spelling out that I do not take each individual’s narratives to be representative of their national identity, be it “Anglo‐Italian,” “Scottish,” or “English.”

By analyzing the specifics of these individuals’ accounts I hope to complicate and challenge entrenched media and social scientific generalizations about who the white working classes are in England, and what their views on immigration and multiculturalism are (see also Tyler, 2015). Since the 2008 recession, the identity of “white working class” has become especially politically laden in popular culture and academia in the UK (e.g., Goodhart, 2017), France (e.g., Guilluy, 2019) and the USA (e.g., Hochschild, 2016). Some sociologists, anthropologists, and political theorists have argued that one aspect of this process in Britain has been the mistaken construction of the white working class as a homogeneous category of people who are routinely constructed in public discourse as “left‐out” and “left‐behind” of cosmopolitan multicultural society, and who are said by some academics, the media, and politicians to hold xenophobic and racist attitudes on immigration and ethnic diversity (see Bhambra, 2017; Flemmen & Savage, 2017; Lawler, 2012; Lawrence, 2019 for critiques of this narrative). This is now a popular discourse in the UK that has become entrenched in public life in the face of Brexit, whereby white working‐class Britons and particularly older generations have become associated in the media and wider political discourse with the nationalistic, racist, and xenophobic undertones identified with aspects of the Leave campaign (see further Whitehouse, 2019, parts 1 and 2, in conversation with me and Degnen about our research on Brexit, identity, and belonging which sets out to interrogate, complicate, and challenge this and related public discourses on Brexit). Significantly, then, my returning to the particular experiences and personal expressions that constitute ideas of home and intercultural conviviality in older white working‐class people’s accounts in the face of these contemporary Brexit narratives illustrates the absurdity of reducing the complexity of individuals’ views on matters of immigration and multiculturalism to a grand singular classed, generational, and racialized narrative.

## 4 GUIDO AND HIS SON MATTEO

When we met Guido (all names are pseudonyms), he was in his sixties, and his son Matteo was in his thirties. They both self‐identify as “Anglo‐Italian.” Guido settled in the town with his wife in the early 1960s from a village near Naples. Southtown offered them the opportunity for work and financial stability which was not possible in the area of Italy in which they lived. Both Guido and his wife were retired. Guido used to work at a local hospital and also worked part‐time as a gardener. At the time of the research, Matteo worked as a football coach at a local youth club and had lived in the town since birth. The research fellow and I met Guido, his son, and his wife at Guido’s and his wife’s home one evening. Guido’s wife was an active listener in the conversation but did not say very much. In what follows I shall explore the differing ways in which Guido identifies with the English nation and the town as “home.”

I turn to how Guido remembered his experience of abuse and black people’s experience of racism when he first settled in the town, and how in spite of this experience England has over time become his home. He said:

I never had any trouble … except … in those days [with] the …. teddy boys …. They were after the foreigners, mostly … blacks…. We were lucky [e.g., Italians]…. They [e.g., black people] were very unlucky, they can spot them…. Apart from that … we start mixing [e.g., with the white English] …. Especially after my children started having English friends and they got to know their friends, their parents and it changed completely. So we’re thinking we’re English…. England started to feel like home … in the first few years when I was going back to Italy on holidays, I felt … I’m coming home. Now it’s the other way round. I go [to Italy] for a couple of weeks … when I come back [to Southtown] I feel I’m coming home.

For Guido it is his white racial identity that has in part facilitated his sense of fitting into the town in spite of his experience of abuse for being identified as a “foreigner.” In other words, Guido’s identification with the town is merged with a sense of inclusion into the category of white Englishness, in a way that was not possible for black people. This feeling of the town and England as home gradually became apparent to Guido through his routine visits to Italy. These visits over time facilitated his realization that Italy didn’t offer the same degree of stability and familiarity that he had in Southtown. Guido suggests that coming to “know” English families through his children’s relationships was central to this process of identifying with the town and the nation as home. I shall explore the significance of “knowing” further in my analysis of Mary’s account.

Later on in our discussion Guido and Matteo emphasized the importance of owning a house to feel at home, and in so doing they elaborated on their relationships with “working‐class English” people. In what follows the idea of home as a site of stability and ontological security comes to the fore. It is worth noting that when they sometimes referred to “working‐class” people and/or “English” people, they mean *white* working‐class English people:

*Guido*: My brother‐in‐law…. He was English…. He was living in a council flat [local authority owned] which when … Thatcher [British Prime Minister from 1979–1991] did the right to buy your own … house…. I was telling him: “look why don’t you buy your own flat…? One day it’s yours ….” And his attitude was “…but if anything goes wrong … ”. He started getting the message … and he applied for it and suddenly … he … died…. So my sister … she bought her own flat…. And that’s the best thing … you can do.

*Matteo*: A lot of friends … they’re in the same position…. Although they can afford to buy a house … the English friends they’re renting…. The working class ones … they still …. don’t think long term…. The house is a pension …. We were expected to save … and come a point in your life … to buy your own house…. I think with my English friends it was ….. different because …. a lot of them had to leave home at a very young age … sixteen plus…. It’s the same … in old age … we’re expected to look after our parents … not put them in a nursing home…. I think the Asian community are very similar…. Whereas my English friends they say that … a lot of their parents are …. happy to go to a nursing home…. But I think it’s just tradition in Italy to be … close to your mum and dad…. Although in Italy it’s changing more … than it is for the Anglo‐Italians here [in England]…..

For Guido and Matteo, the feeling of familial warmth identified with being at “home” is expressed through the stability and security that comes with owning a house. To explain the value that they invest in owning a house, Guido and Matteo position themselves as “Anglo‐Italian.” This is an identity that captures the ways in which the “borders of Italian and English cultures blur” in Guido’s sense of belonging to Southtown and the nation as “home” (Fortier, 2000, p. 163), but this is also an identity that takes meaning in contrast to “the working‐class English.” They think that “Anglo‐Italians” own their homes—a process that parents actively support their children in achieving because it brings financial stability and security. In this way, “Anglo‐Italian” families are thought by Guido and Matteo to be guided by an ethics of care that encourages children to stay close to their parents throughout their lives. In contrast, “the English working classes” are thought by Guido and Matteo to be concerned with an ethics of individualism and independence. In this scenario home ownership is considered to be a burden of responsibility. Moreover, children and parents are thought to actively seek independence from each other. Clearly, the ideals attached to the practice of home ownership inform what is perceived to be a differing set of working class “English” and “Anglo‐Italian” moral, ethical, and social practices, values, and worldviews. In this way, then, for Guido and Matteo, owning a house is seen as central to the “security of home … knowing what to expect and that the expected is likely to occur” (Case 1996, p. 11 cited in Skey, 2011, p. 245).

To draw out further Guido's and Matteo's feelings of connectedness to and distinction from “working‐class English” people, it is useful to compare their account with Benson and Jackson’s (2017) analysis of home ownership and the performance of middle‐class identities in London. They argue that for young middle‐class professionals, the inability to own a property due to the inflation of house prices is emotionally demoralizing. This is because home ownership is felt to be essential for the reproduction of middle‐class subjectivity and status. My contention is that for Guido and Matteo, home ownership is not about aspiration for middle‐class status. Rather, for them home ownership is important for the reproduction of “Anglo‐Italian” values, tradition, security, stability, familiarity, and identity centered upon ideals of home. These “Anglo‐Italian” values and practices hold the potential to be appropriated by “English working‐class” family members such as Guido’s brother‐in‐law. In this regard, a “fault line” cannot easily be “drawn” between people according to ideas of identity and national difference (Heil, 2014, p. 463). Rather, “working‐class English” people’s conformity to what are perceived to be “Anglo‐Italian” values, encapsulated in the practice of home ownership, become entwined with the formation of intercultural relationships of friendship and kinship.

Significantly, Matteo positions “Asians” as family oriented like “Anglo‐Italians.” However, Guido prefaced his description of “Pakistanis” (his term) that reside in the town with the following comment: “…they don’t integrate, they like to form themselves a ghetto and then they blame us.” To elaborate on his perception of “Pakistani” residents, Guido reflects on his experience of living in a street situated near to the town’s mosque. This neighborhood is known locally for being the home‐place of British Asian Pakistani Muslims. Guido and his family lived in this area for 27 years. Guido explained his recent experience of selling his house in this neighborhood as follows:

You know [when] every English, Spanish or Italian moved out … a Pakistani move in…. When we wanted to buy a bungalow … I had difficulty in selling the house, difficult …. because of them…. We had the house on the market for a couple of years. It’s only the Pakistani came to see the house, no other nationality. I mean they just create themselves a ghetto….

*Matteo*: It was a road which was predominantly mixed.

*Guido*: Pretty mixed…. There wasn’t any trouble you know with any one of them.

*Researcher*: But the Italian families knew each other and the Pakistani families knew each other?

*Guido*: They knew each other, that’s right … but they keep their distance.

*Researcher*: So they never became like friends?

*Guido*: No, no I tried you know…. I don’t think anyone moved because of the Pakistanis…. It was because of family situations…. Although they create a bit of difficulty if you want to sell a house.…

Guido and Matteo paint a picture of their street whereby “Italian,” “Spanish,” “English,” and “Pakistani” neighbors live side by side, although they did not become friends. In this way, they convey a general feeling of pragmatic indifference (Wessendorf, 2013) towards perceived national and racial differences, especially when there “wasn’t any trouble.” It is in this spirit that Guido and Matteo are keen to stress that no one moved out of the street because they did not want to live alongside “Pakistanis.” However, Guido did think that the numbers of “Pakistanis” that lived in the neighborhood hindered the sale of his house because the only buyers were “Pakistanis.” In this instance Guido’s pragmatic feelings of indifference towards his “Pakistani” neighbors slide into a feeling of frustration with “them.” This feeling of frustration is expressed in the language of commonplace stereotypes about British Asian Muslims in the UK. For instance, Guido repeats his experience that “Pakistanis” are responsible for creating a “ghetto.” The term “ghetto” is a racialized and derogatory term often used in public discourse in the UK to refer to urban areas where ethnic minorities and migrants live. This term also evokes the commonplace Islamophobic image that British Muslims, usually associated with people of South Asian descent, are leading “parallel lives” to the rest of British society (Philips, 2006; see Casey, 2016 for a recent policy reiteration of this narrative). For Guido, “Pakistanis” should be accepted when *they* express proper neighborly behavior and invest in the local neighborhood (see Erel, 2011, p. 2054). In this way, then, Guido is acquiring “especially British competencies” of speaking and thinking about British Asian Muslims, and so positioning himself “into Britain’s racialized status hierarchies” as one of “us” rather than one of “them” (Fox & Moglinicka, 2019, p. 5). These racialized stereotypes about British Asian Pakistanis become meaningful for and take hold of Guido when he thinks the stability and security that comes from home ownership is under threat. My contention is, then, that we can only understand the full force of Guido’s anxiety, which is created by the fear of not being able to sell his house, if we take into account the way in which home ownership is central to the reproduction of his family’s sense of well‐being, “Anglo‐Italian” identity and ontological security.

I now turn my analytical attention to Mary’s narrative. Mary’s relationship with her British Asian neighbors is mediated by a different construction of home to that harbored by Guido and Matteo. Nevertheless, she does share Guido’s and Matteo’s aspiration for stability and security.

## 5 MARY

When we met Mary she was 59 years old and had lived in her current flat (e.g., apartment) that she rents from the council for some forty years. Mary was born in Scotland, but like Guido has lived in Southtown for most of her adult life. She moved to the town from Scotland when she married her husband. Mary has three adult daughters who have left home.

Mary is reluctant to move out of the three‐bedroomed flat that she lives in with her husband, even though she thinks the council—from whom she rents the flat—would like her to move. As she said: “they would give their eye teeth for a three bedroom flat,” thereby highlighting the shortage of council‐owned property in the town. This is a well‐documented consequence of the British government’s policy since the 1980s of selling council houses to tenants. Some tenants that bought their houses, like Guido’s sister, greatly benefited financially from the right to buy scheme. However, councils across the UK have not replaced the sold‐off housing stock. This policy therefore led to a chronic shortage of council housing. This has had devastating consequences for families in need of subsidized rental housing both locally in the town and nationally.

While Mary is aware of this shortage of council properties in the town she says: “I like living here and if I didn’t … I would move.” By contrast to Matteo and Guido, Mary has no desire to own her home. Mary considers home ownership in the block of flats in which she lives deeply problematic. This is because the council are not responsible for the maintenance of privately owned properties. Consequently, Mary thinks that there is no guarantee that the flats in her building are properly maintained and thus safe. For Mary, the council is responsible for making her home safe and secure. By contrast, Guido and Matteo think that renting from the council thwarts families’ chances of financial security and social stability obtained through ownership.

It is also significant that Mary is not emotionally attached to her flat. One of her daughters described her flat as “very kind of neutral…. There’s …no sign … of who lives there.” Nonetheless, Mary does feel a strong sense of responsibility and emotional connection to the people who live in the other flats in her building. This connection is formed through her role as secretary of the local residents’ and tenants’ association. Together with a British Asian neighbor, Mary plays a key role in representing the interests of both leaseholders and tenants that live in her building to the council. She described how this role has shaped her relationships with her “Asian” neighbors as follows:

They come and knock on my door when they need anything. Like downstairs have had a problem, they … ring my doorbell…. Upstairs, they’re Asian, they will knock, come down and speak to me. You know if there’s a problem…. If I come with my shopping and the children are out … they will bring my shopping up…. We do it for them if they’ve got a lot of shopping. I will help them….

In stark contrast to Guido’s relationship of indifference towards his “Pakistani” neighbors, Mary depicts a mutually warm relationship between herself and her “Asian” neighbors. This relationship is formed through her daily interactions with them that give her a feeling of familiarity, security, and warmth. It is also clear that the relatively easy ways in which children form relationships with each other and adults plays a key role in the daily maintenance of Mary’s convivial relationships with her “Asian” neighbors. This resonates with the importance that Guido puts on his children’s relationships with “English” families in the shaping of his feeling for the town and England as home. Mary continues by explaining the specific character of her relationships with her female “Asian” neighbors thus:

I do *know* what the [Asian] ladies will do …. I wouldn’t dare say to them: “come to my meeting tonight …” but I would say: “I’m having something outside, would you like to come?”…. I’ve sat at council meetings and they’ve said: “oh we’re going to ask the Asian ladies to do this”. And I’ve said “yeah, OK” [cynical tone]. “Oh don’t you think they can do it?” I said “no I *know* they can do it, but they’ll choose not to….” They say: “well you *know* everything”. I said: “it’s not a question of *knowing* everything, it’s a question of *knowing* [what the Asian ladies will do]….” At one stage the Asian ladies wouldn’t talk to you at all…. But now … when, I went for a meal at the Mosque … all the ladies cuddled me. Now they would never do that before, but they do now, they give me a cuddle [*my emphases*].

Mary repeats that she “knows” the “Asian ladies.” It is also worth recalling that in Guido’s explanation of how England and Southtown have come to be home for him, he emphasized how his children came to “know” English families. Degnen (2013) has eloquently written about the meaning of “knowing” in a village in the north of England where she conducted ethnographic fieldwork. For according to Degnen (2013, p. 555), “knowing is about more than a familiarity with, information acquired, or social networks, as might be commonly assumed. This is because knowing is often evoked by people when seeking to explain what it is that matters in the webs of relations binding them to others and to where they live.” I would like to suggest that it is some of the weight of this kind of knowing that Mary evokes in her description of her relationships with her “Asian” neighbors. Her relationships of affection and trust with the “Asian ladies” formed over time means that she has come to “know” them, in a way that council representatives do not. It is this sense of “knowing” her female “Asian” neighbors that makes Mary feel close to and connected with them, and in turn contributes to her feeling of certainty, stability, and familiarity that makes this place her home.

However, an important caveat needs to be registered here. For the people that feature in Degnen’s study, knowing includes fine‐grained details about individuals’ and families’ histories in the village, its landscape and each other, accumulated over time and across generations. In contrast, Mary’s way of knowing “Asian” women facilitates a sense of connection to them as a group of people that are experienced as acting and behaving collectively. In this way, her representation of her “Asian” neighbors is reminiscent of the commonplace media and policy orientated stereotypes about the supposedly collectivist and gendered constitution of Muslim Asian “culture.”

But yet to end my analysis here would be to diminish the significance of Mary’s mutually warm, friendly, and familiar relationships with “Asian” women formed over time, and the ways in which her sense of knowing “them” is a source of happiness and pride for her that contributes to her feeling of being at home in this place. There is, then, not simply a division of the world into “us” and “them,” but a merging of racialized stereotypical *knowledge* about “the Other” across perceived ethnic, racial, and religious differences, and the colloquial English way of *knowing* people and a place through shared and embodied neighborly relationships and histories.

In the light of these considerations, my question now becomes: how does tracing the everyday construction of home as a stable and familiar space facilitate insight into the circumstances in which the relationship between “Pakistani” and white neighbors is thought by some white residents to break down beyond repair?

## 6 BETTY

Betty is English, in her seventies, and moved to the town from London 24 years prior to the fieldwork in order to be near her brother after her husband had died. She had lived in central London, where her husband worked as a gardener. The research fellow and I met Betty at her house and she described to us how she found her house in the town:

I fell in love with this [house] …. because there’s no houses in front and I’ve got the canal … and the green…. I liked it because I love gardens….

Throughout our conversation Betty returned to the difficulties she said she was experiencing with her “Pakistani” and “foreign” (her terms) neighbors. She told us that her neighbors were disrupting the tranquillity that she associates with suburban life:

When I first moved … here … the lady next door … she was a sweet lady. She was always in her garden…. She had to go into a [nursing] … home and her son sold it [e.g., the house]. But unfortunately it’s [now] not very nice next door…. Pakistanis bought it and they let it out in rooms…. Now there’s a family of three children in one room and another family of three children in another room…. Unfortunately they’re driving me round the bend [e.g., mad]. I’ve been … thinking of moving…. They just throw their rubbish … in the garden and I’ve started having rats out there…. [There is] a lot of noise at night … banging and… children shouting…. I’m not racist but it’s very annoying when … I’ve come down here [from London] to have a bit of peace and quiet and it was lovely when I first moved here and it’s not now…. When I was young … one of my aunts … she said … “in years to come … the foreigners will take over this country”. I know I shouldn’t say it, but … it’s not our country any more…. I think you’ll find a lot of elderly people will tell you the same…. But [we’ve] just got to put up with it now I’m afraid…. I don’t think it’s right that a family of a man, a woman and three children, young children should be in one room. I think it’s disgusting…. I know children have to play, I mean fair enough, but … sometimes at 12 o’clock at night they’re out there shouting and hollering. They should be asleep…. Because … their hallway is behind that wall [pointing to a wall in her hallway] and they’re running up and down the stairs … banging up and down the stairs. It’s … terrible…. You can’t relax and go to sleep…. And the garden … it’s a jungle.

Betty does not make any distinction between “Pakistanis” who settled into the area in the 1960s and newer migrants. Rather, her neighbors are positioned collectively as “foreigners” who she thinks represent a threat to the peace, respectability, and security of her suburban home and garden. Echoing Guido’s and Matteo’s ideals of home, Betty identifies a sense of being at home with the security and materiality identified with owning a house. Her sense of security, emanating from her house and garden, she thinks is destroyed by her neighbors. To her, her migrant neighbors represent a destructive force who she thinks have contravened the accepted norms of neighborliness. These norms are exemplified for her by the way her former neighbor used to maintain her garden. Her current neighbors have, she says, not only ruined the aesthetics of her garden, but have also interfered with her sleep and peace of mind. This broken relationship forms a contrast to Guido and Matteo’s pragmatic relationships of indifference to their “Pakistani” neighbors, and Mary’s mutually warm relationships with her “Asian” neighbors. While Betty thinks it is “disgusting” that the migrant families live in one room, her misery hinders her from feeling sympathy for the hardships these families endure.

While Guido entwines his identification of the town as home with a sense of the English nation as home, it would seem for Betty the reverse is true. Thinking of her neighbors, Betty is reminded of her aunt’s prediction that “foreigners” will “one day take over this country.” The felt destruction of the stability, familiarity, and security of her home is mirrored in her perception of a detrimental impact of immigration on the nation. But yet, Betty is also wary that others might identify her views as “racist,” an attribution she explicitly disavows. In this regard, she told us that her views would get her “into trouble,” possibly with younger people like myself, and the research fellow. It is with this concern in mind that she pre‐emptively defends her position by suggesting that many “older people” would agree with her.

In sum, then, Betty’s reported experiences once again exemplify how everyday ideas of home encompass feelings of familiarity and security associated with neighborly relations and the materiality of owning a house. My contention is that special attention to this construction facilitates insight into how an abstract notion of the “foreigner” becomes real and is thought to represent an attack on the very fabric of one’s self, home and nation.

## 7 CONCLUSION

My argument in this article is that special analytical attention to everyday constructions of home in a suburban town has the explanatory power to deepen current sociological analysis of the co‐existence of relationships of intercultural conviviality‐and‐racism. That is to say, most studies working within the everyday multiculturalism and conviviality paradigm tend to focus on formations of urban diversity in more public and semi‐public sites of interaction, such as cafes, parks, and leisure spaces, in super‐diverse cosmopolitan cities. While home space is integral to these studies, everyday constructions of home are not given the special analytical attention that they deserve and so are under‐researched as sites of conviviality‐and‐racism. Moreover, as Vincent et al. (2018) argue, the interconnections between public and semi‐public places and home spaces as sites of interethnic conviviality are not fully explained in this literature. In this article, I have sought to redress this oversight. In so doing, I have traced the myriad ways in which home emerged as a dominant site through which people spoke about their relationships across ethnic, national, and racial differences in our study of an English suburban town. Given the significance of the idea of home to the imaginary of English suburban life, it is not surprising that home emerged as a focus for the formation of interethnic relationships.

In this article, then, I have traced the diverse ways in which ideas of home become associated with ideas and feelings of stability, familiarity, and security. From this point of view, home space becomes identified with a diverse array of material and ethereal aspects of social life that intersect the public, semi‐public, and private spheres, including: the materiality of owning a house; the tranquillity and respectability of suburban life; the norms and values of neighborly relationships (for example, help with shopping, not getting too involved in each other’s lives and maintaining a tidy garden); the sense of *knowing* your neighbors; the intergenerational warmth of being part of a family and identification with the town, and ultimately with the nation as home. I have highlighted the varied ways in which British Asian Pakistani residents across perceived national, religious, racial, and ethnic differences can become incorporated into and constitutive of these relationships, values, and feelings associated with home and home space. My analysis also highlights how the social fabric of home includes interethnic relationships formed in public and semi‐public spaces, for example, at the mosque, at the local residents’ association and in daily interactions between neighbors in shared social spaces such as the street and apartment buildings. But yet, also crucial to my analysis is an exploration of those instances when a breakdown in the ontological security, warmth, and familiarity associated with home can open up a space for commonplace racialized and racist stereotypes of difference to take hold. In this regard, my analytical attention to everyday constructions of home has enabled me to trace how widely held racialized stereotypes about British Asian Pakistanis’ supposedly collectivist lifestyle and perceived lack of integration into the neighborhood and nation become salient. In this way, then, the ideas, feelings, relationships, and values associated with home and home space become entwined with articulations of conviviality‐and‐racism within the neighborhood, the town, and ultimately the nation.

One might wonder, is there antiracist potential of this study? I do not want to put too much weight on how the formation of warm intercultural relations of neighborliness, friendships, and sociality reported here are transformative in terms of fostering everyday antiracist consciousness and action, as some scholars of conviviality studies have been criticized for doing. Nor do I want to dismiss the voices of the people that we hear in this article as simply prejudiced, as some critics of the conviviality study’s approach might conclude. My suggestion is that something much more complex is at work here. That is to say, those moments in which people considered to be ethnically, nationally, and racially other become positioned and included into the warmth, neighborliness, security, and familiarity of white working‐class people’s homes offers an important counter‐narrative to the sometimes panicky public and academic debate about the “creeping fascism” infiltrating Brexit‐laden British society. Interpreting this interview data gathered some 10 years ago through the lens of current public and political debates on Brexit complicates and challenges any straightforward association of anti‐immigration, xenophobic, and racist attitudes with the white working classes and particularly older generations of people. Furthermore, Guido’s blurring of England and Italy as home problematizes any xenophobic idea that Europeans living in the UK after Brexit are in some way “foreigners” and “outsiders” to Britain. Notwithstanding the significance of these insights, my study also shows how racialized stereotypes, particularly those about British Asian Muslims, and “foreigners” in general, are never too far out of reach. Such stereotypes of racialized difference are so ingrained in the postcolonial, institutional, and social policy fabric of British society that they easily take hold in this suburban town, especially when daily relations between neighbors are thought to intrude on the security and familiarity associated with home and home space. It is precisely the open and closed constitution of interracial and interethnic relationships that underpins the reproduction of everyday racism and multiculturalism not only in the home spaces of this suburban town but also, I would suggest, within everyday constructions of home and home spaces across the Western world.^3^

## NOTES

1 This research formed part of an ESRC funded project (RES‐000‐22‐2796): “Communities within communities: a longitudinal approach to minority/majority relationships and social cohesion.” Ole Jensen was the project research fellow, and I was the principal investigator.

2 I am currently the principal investigator of an ESRC funded research project that is exploring these questions of Brexit, race, ethnicity, nationhood, migration, class, and generation. The project is called: “Identity, Belonging and the Role of the Media in Brexit Britain” (ES/R0005133/1). For information on the project and the team see: //www.brexitandbelonging.org/://www.brexitandbelonging.org/

3 I would like to thank: Sarah Heinz (University of Vienna), Anouk de Koning (Leiden University) and Charles Leddy‐Owen (University of Portsmouth) for each inviting me to present the ideas in this paper at workshops that they each organised; Cathrine Degnen for insightful discussions about knowing, as well as Brexit that forms our current work; Nigel Pleasants and three anonymous referees for their careful reading and insightful comments on versions of this article.
